# Recent Advances in Plasma-Engineered Polymers for Biomarker-Based Viral Detection and Highly Multiplexed Analysis

**DOI:** 10.3390/bios12050286

**Published:** 2022-04-28

**Authors:** Seyyed Mojtaba Mousavi, Seyyed Alireza Hashemi, Masoomeh Yari Kalashgrani, Ahmad Gholami, Navid Omidifar, Aziz Babapoor, Neralla Vijayakameswara Rao, Wei-Hung Chiang

**Affiliations:** 1 Department of Chemical Engineering, National Taiwan University of Science and Technology, Taipei City 106335, Taiwan; vijayrao@mail.ntust.edu.tw; 2 Nanomaterials and Polymer Nanocomposites Laboratory, School of Engineering, University of British Columbia, Kelowna, BC V1V 1V7, Canada; s.a.hashemi0@gmail.com; 3 Biotechnology Research Center, Shiraz University of Medical Sciences, Shiraz 71468-64685, Iran; masoomeh.yari.72@gmail.com (M.Y.K.); gholami@sums.ac.ir (A.G.); 4 Department of Pathology, Shiraz University of Medical Sciences, Shiraz 71468-64685, Iran; omidifarn@sums.ac.ir; 5 Department of Chemical Engineering, University of Mohaghegh Ardabil, Ardabil 56199-11367, Iran; babapoor@uma.ac.ir

**Keywords:** viral detection, plasma-engineered polymers, highly multiplexed, biomarkers

## Abstract

Infectious diseases remain a pervasive threat to global and public health, especially in many countries and rural urban areas. The main causes of such severe diseases are the lack of appropriate analytical methods and subsequent treatment strategies due to limited access to centralized and equipped medical centers for detection. Rapid and accurate diagnosis in biomedicine and healthcare is essential for the effective treatment of pathogenic viruses as well as early detection. Plasma-engineered polymers are used worldwide for viral infections in conjunction with molecular detection of biomarkers. Plasma-engineered polymers for biomarker-based viral detection are generally inexpensive and offer great potential. For biomarker-based virus detection, plasma-based polymers appear to be potential biological probes and have been used directly with physiological components to perform highly multiplexed analyses simultaneously. The simultaneous measurement of multiple clinical parameters from the same sample volume is possible using highly multiplexed analysis to detect human viral infections, thereby reducing the time and cost required to collect each data point. This article reviews recent studies on the efficacy of plasma-engineered polymers as a detection method against human pandemic viruses. In this review study, we examine polymer biomarkers, plasma-engineered polymers, highly multiplexed analyses for viral infections, and recent applications of polymer-based biomarkers for virus detection. Finally, we provide an outlook on recent advances in the field of plasma-engineered polymers for biomarker-based virus detection and highly multiplexed analysis.

## 1. Introduction

One of the leading causes of diseases that kill hundreds of thousands of people every year is the contamination of sources by viruses. As millions of people suffer from various diseases, these medical problems have not yet been solved [1]. Nowadays, there is a significant increase in infectious diseases that have a significant impact on all living species, such as humans, plants, and animals [2]. Especially in many countries and in the poor strata of modern society, many people are affected by various infectious diseases such as influenza, coronavirus, and human immunodeficiency virus, which continue to cause significant health problems [3]. Viruses are intracellular parasites and require a host cell to replicate genetic material. In response to very rapid changes in complex protective mechanisms, the host immune response is manipulated, and viruses adapt by refraction. This has led to the emergence of viruses that manipulate host safety responses and have compatible subdomains. In addition, viral infections cause deaths worldwide. Outbreaks of the Ebola virus in 2014 and influenza A H1N1 in 2009 have attracted attention in recent years [4]. The early detection of pathogens such as bacteria and viruses is vital for clinical care [5]. A biomarker is a biological molecule used as an indicator of the occurrence of a certain biological condition or stage, such as the presence of a microorganism or the occurrence of a disease or biological process, the stage of the disease, and some other important cases. In some studies, biomarkers can be used as key molecules to identify metabolic pathways, message transmission, and so on. Biomarkers can also facilitate the molecular definition of a disease or provide useful information about the different stages of a disease, or the early diagnosis and susceptibility of individuals to different diseases, as well as predict the cellular response to a therapeutic drug. Biomarkers can consist of various molecules, such as proteins, nucleic acids, and other metabolites. Biomarkers have been used for the early detection or prognosis of diseases, especially infections. The detection of biomarkers for disease diagnosis can be performed by measuring enzyme activity, detecting specific antigens, and detecting specific nucleic acids by conventional methods such as IHC, PCR, and ELISA. The ideal biomarker for disease diagnosis should have characteristics such as high sensitivity, high specificity, and high accuracy in indicating disease states, and in the case of infectious diseases, it should also be able to predict the outcome of treatment [6,7,8,9].

Highly multiplexed analysis is used for the rapid detection of nucleic acids, which includes the detection of infectious diseases, biomarkers, and viruses. The introduction of highly multiplexed analysis has greatly improved the quality of the clinical microbiology laboratory and the detection of viruses based on biomarkers, and has been used as a tool for the diagnosis of emerging diseases, such as new influenza viruses and coronaviruses, in diagnostic tests [10,11,12,13,14]. Since controlling the surface properties of polymers is essential to improve their performance, this has led to the application of these materials in medicine. The behavior of polymers at the surface compared to the bulk is different. The reason for this can be related to the surface effect, which is caused by the asymmetry of forces acting on a molecule at the surface. On the one hand, in soft materials such as polymers, the surface effect causes the formation of chains on the surface to change. On the other hand, polymers have low surface energy due to the weakness of the molecular bonds between chains. For this reason, surface correction should be performed to improve adhesion and wettability properties. This surface modification involves the surface absorption of molecules with the properties of polymer planes in order to change the properties of the polymer surface [15,16,17,18]. One method of surface modification involves plasma. Plasma formed from atoms, molecules, and radicals is also called the fourth state of material [19]. About 99% of the materials in the world are plasmas. The strong electric field generated between two electrodes causes gas ionization between two electrodes and creates a mixture of ions, atoms, electrons, and molecules, which is called plasma. In fact, plasma is a mixture of pregnant parts and neutral atoms. Therefore, plasma has a higher energy level compared to other materials, whether solid, liquid or gas. Plasma has a chemically active and unusual environment in which many surface reactions take place. The high density of ionized and stimulated constituents in plasma can change the surface properties of an inherently neutral material, such as ceramics and polymers. Surface modification with plasma can change a polymer’s adhesion, hardness, chemical neutralization, surface wettability and biocompatibility. The advantages of using plasma for surface modification include reliability and reproducibility, cheapness, applicability to various shapes with geometric complexity of the surface, such as felts and fabrics, uniformity of correction, and the possibility of patterning for correction [20,21,22,23,24]. Plasma-engineered polymer-based biomarkers that were developed to detect biomarkers of viral diseases are shown in Table 1.

The aim of this review study was to review the recent advances in plasma-engineered polymers for biomarker-based viral detection and the highly multiplexed analysis of the work in this field. In addition, polymer biomarkers, plasma-engineered polymers, highly multiplexed analysis for viral infections, and recent applications of polymer-based biomarkers for virus detection were evaluated.

## 2. Polymer Biomarkers

**Figure 1:**
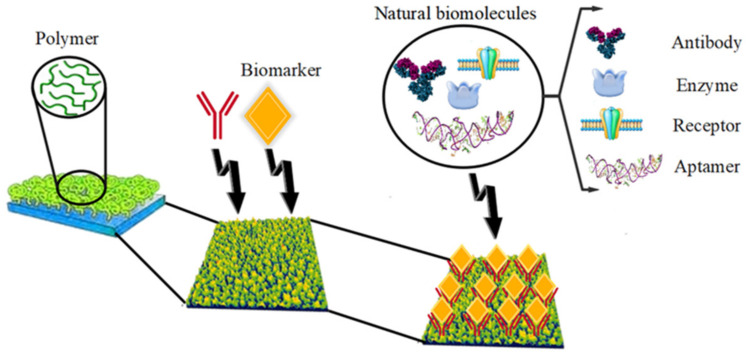
Polymer biomarkers.

Factors such as rapid, ultrasensitive, and noninvasive biomarker diagnosis are important for infectious-disease detection. According to studies, natural biomolecules such as aptamers, antibodies, receptors, and enzymes act as sensors to detect infectious biomarkers [36]. Enzymes such as proteins and nucleic acids (called ribosomes) remain unchanged during the completed reaction; therefore, they can continue to interact with substrates. Enzymes can increase the rate of reactions by millions of times during a given process. Enzymes can be affected by molecules that increase their activity, “activators,” or by molecules that decrease their activity, “inhibitors” [37,38]. Antibodies are specific proteins that detect foreign substances in the blood and help the body remove of them [39,40]. Therefore, in recent decades, the identification of infectious biomarkers based on polymeric materials compared to natural biomolecules (because of stability and high cost) has attracted much attention. The polymer biomarkers are shown in Figure 1. Since polymers have similar sensitivity to natural receptors, they are also referred to as “synthetic antibodies.” Polymers show great potential as artificial detection elements due to their better chemical and mechanical stability in biomarkers. Following the simple “lock-and-key” mechanism, polymers can be prepared that first allow polymerization around the pattern molecules. The pattern is then removed to create artificial junction sites [41,42]. Therefore, polymer biomarkers must be structurally complementary to each other. However, to identify polymer biomarkers, in many cases, it is not sufficient to determine the size and shape of the complement. Chemical binding sites similar to those found in natural target–receptor interactions must also be formed. Creating high selectivity and binding sites for target hosts is possible by following this principle and selecting the right number of monomers. During the polymerization step, chemical interactions play an important role in the shape and size of the polymer biomarker additions, and even in the prepolymerized mixture, these interactions can be covalent or noncovalent. Thus, in a covalent interaction, the mold and the functional monomers (oligomers with one, two, or even more functional end groups such as -OH, -COOH, -NH_2_, -SH, etc.) are initially linked by covalent bonds, and these bonds are chemically broken when the template is removed. In contrast, in a noncovalent interaction, the mold and the functional monomer interact only through reversible noncovalent bonds (such as hydrogen bonds, electrostatic interactions, and van der Waals), and these noncovalent interactions can be reversed by washing the polymer biomarkers with a suitable solvent [43,44,45,46].

## 3. Plasma-Engineered Polymer

**Figure 2:**
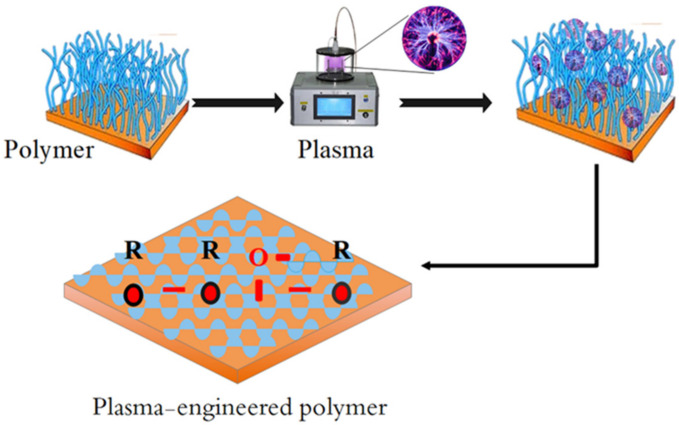
Plasma-engineered polymer.

In the selection of plasma-engineered polymers according to their purpose and final application, numerous factors such as the size of the nanoparticles in question, the properties of the material (aqueous solubility, stability, etc.) to encapsulate in the polymer, surface characteristics, and performance depend on bioavailability, biocompatibility and yield characteristics of the final product [47,48]. Polymers engineered with selective plasma must be biodegradable, have a specific degradation rate, have good adsorption, have a controllable and cost-effective release, be easily attached to the active ingredient, and not be subject to biological activity. In general, the use of plasma on polymers can be divided into two categories: The first category is the surface modification of polymers, which can refer to the hydrophilicity or hydrophobicity of polymer surfaces. In this method, it is possible to remove weak bonds on the surface of polymers and create new bonds. The smoothness of the surface can also be controlled [49,50,51]. Figure 2 shows plasma-engineered polymers. The above method is used for the detection of biomarkers of viral infections and antibacterial treatment of medical device surfaces. The second category is plasma-assisted polymers, gas monomers and their deposition on the substrate. Sometimes plasma sputtering is used in this method [52,53,54,55,56]. An example of this method is coating with a thin layer of organic polymers as dielectric, which improves its properties [57]. Binding of the enzyme to various polymers such as albumin, dextran, polyethylene glycol, chitosan, silk fibrin and sericin has been done, which has led to the improvement of the physicochemical properties of the enzyme [58]. Additionally, these plasma-engineered polymers have been extensively studied for the delivery of pharmaceutical proteins and antigens due to their adhesion properties and increased adsorption [59]. Therefore, in the process of attaching plasma-engineered polymers to enzymes, the polymers or enzymes have limited active groups. Additionally, in order to attach the enzyme to synthetic or natural polymers, activation of groups on the enzyme or plasma-engineered polymers is essential. The type of activated derivatives has a great impact on the structure and consequently the function, activity, stability and immunogenicity of the enzyme [60]. The results of research related to the stabilization of plasma-engineered polymers to proteins have shown that if they are bonded to each other through multiple aldehyde bonds (multi-point bonding), then the stability of the polymer–protein complex will be greater against changes in the spatial structure caused by heat, organic solvents, freezing, melting, and digestive enzymes. Therefore, the use of aldehyde bonds such as glutaraldehyde in the incorporation of polymer-engineered polymers into multiple enzymes has been used and has had good results in improving their performance [61]. The stabilization of plasma-engineered polymers on enzymes has improved the stability of enzymes against temperature, pH and non-optimal storage conditions. However, the process of incorporation of these plasma-engineered polymers into enzymes and their type of chemical structure have a significant effect on the physicochemical properties and stability of the enzymes under non-optimal conditions [62].

### 3.1. Plasma Processing of Surface Morphology for Polymers

**Figure 3:**
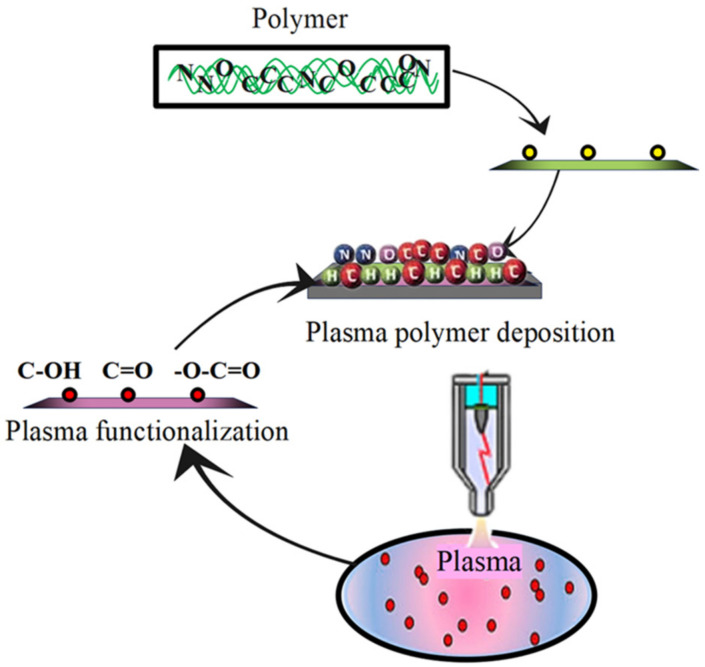
Plasma processing of surface morphology for polymers.

The surface morphology of the polymer can be altered by methods such as heat treatment, irradiation, and plasma, which in the plasma method, in addition to the factors mentioned above, can lead to the breaking of some bonds and the separation of some functional groups from the surface [63,64], some materials can be deposited on the surface, without changing the properties within the polymer volume [65] (film deposition), and/or some new bonds can be created on the surface of the polymer (grafting). Additionally, some new polymer networks can be created on the surface (branched bonds of polymers), and the polymerization process can also be carried out on the surface [66]. Plasma processing of surface morphology for polymers is shown in Figure 3. In general, the effect of plasma on the surface morphology is used to improve the wettability and adhesion of the polymer and to increase the mechanical, chemical, and physical resistance of the polymer for various applications. This effect extends to a depth of 0.05 µm to 0.0005 µm, but leaves the volume inside the polymer unaffected [67]. Favorable plasmas for operations to correct surface morphology are plasmas that discharge electric radiation with alternating voltage [68]. The main advantages of modifying surface morphology with plasma are [69]: (1) It leaves a uniform effect on the surface. (2) It is very fast and saves time. (3) There is no heat damage with this method.

### 3.2. Plasma Processing of Surface Chemistry for Polymers

**Figure 4:**
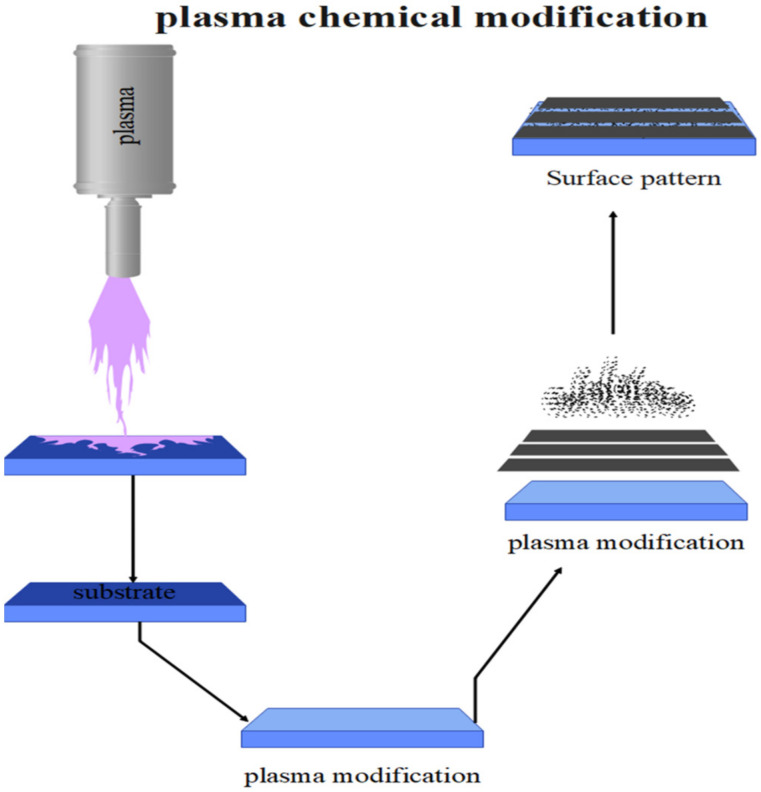
Plasma chemical modification.

As the polymers are sufficiently exposed to the plasma, a decrease in polymer mass occurs, and a layer detaches from the polymer surface. The rate of mass decrease depends on the type of polymer and the energy level of the plasma. Polymers that have oxygen-containing functional groups in their chains, such as carboxyl and ester groups, are very sensitive to plasma. The reduction in mass due to separation is specific to the surface layer, while the inner layers and the polymer mass are hardly affected by this phenomenon. The separation in polymers is mainly due to the failure of the chemical bond in the polymer by the plasma and the reaction with the radicals generated in the polymer chains. Depending on the polymer, these generated radicals can cause the formation of cross-links or the degradation of molecular chains and the formation of products with reduced molecular mass. As the polymers are exposed to the plasma, chemical separation occurs in addition to the physical separation process. Chemical separation is a process that occurs by highly mobile ions (more than ten electron volts). When the ion beam is irradiated onto the solid surface, some of the ions are reflected from the solid surface, and most of the ions penetrate into the matter from the surface. During the diffusion process, the ions lose their kinetic energy due to elastic and inelastic collisions with the matter, and the diffusion is stopped [70,71]. Figure 4 shows plasma chemical modification. Therefore, gases and vapors of monomers are used to modify the surface, which is often accompanied by the formation of functional groups and cross-linking of the surface [72]. These gases include, but are not limited to, argon, neon, helium, hydrogen, ammonia, carbon monoxide, carbon dioxide, oxygen, water, nitrogen, and nitrogen dioxide. The plasma of noble gases leads to the formation of radicals on the surface. The initially formed radicals have a strong tendency to react with other radicals, which in most cases leads to cross-linking of the polymer surface. Moreover, after correction, these radicals lead to functionalization. Thus, after leaving the argon environment, the radicals created by the modification react with the oxygen in the air or with a small percentage of oxygen in the chamber, creating polar groups on the surface [72,73,74]. When the surface is modified with active gases such as oxygen and ammonia, the oxygen-containing and NH_2_ groups, respectively, are bound to the surface. Due to the polarity of these factors, there is a significant increase in acceleration and surface energy in both cases [75,76,77]. The vapor of fluorinated monomers such as SF6 causes fluorinated substances on the surface, which cause surface hydrophobicity [77,78,79]. Magliulo et al. used XPS X-ray photoelectron spectroscopy to study the surface chemistry and chemical changes caused by plasma modification. The results showed that the two peaks of 284.8 and 289.4 indicate the ether or carbonyl oxygen groups and the carboxyl groups formed by the surface modification, respectively [80].

## 4. Highly Multiplexed Analysis for Viral Infections

**Figure 5:**
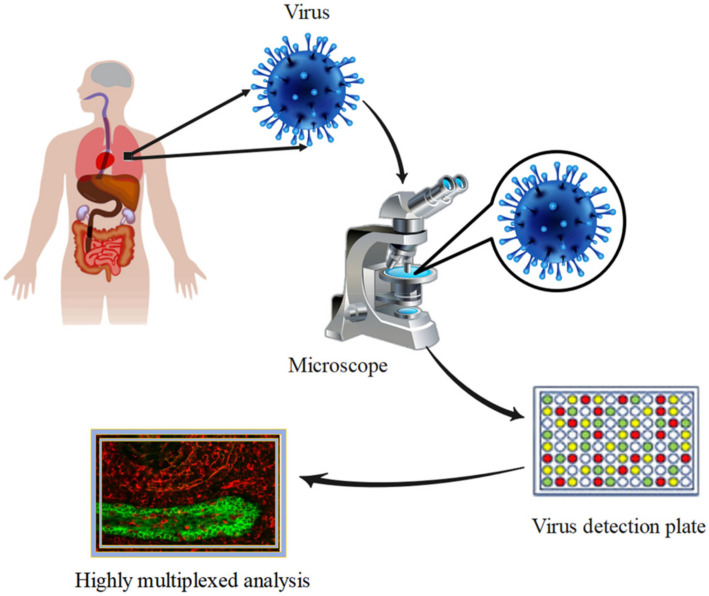
Highly multiplexed analysis for viral infections.

Since the quantitative and qualitative detection of biomarkers is essential for disease diagnosis, biomarkers can be used to objectively evaluate pathogenic processes and biological processes [81,82,83,84]. However, the detection of a single biomarker is not sufficient to diagnose a disease, although a single biomarker may indicate more than one disease [83,85,86]. For example, using a single biomarker to fully detect viral disease in infected individuals can lead to false positives and negatives [87]. The highly multiplexed detection of biomarkers in a single assay has also been used to increase detection accuracy through more accurate scientific information and to improve detection performance through faster analysis in order to avoid misdiagnosis [88,89,90]. Therefore, it is important to clinical detection to develop techniques for the highly multiplexed detection of disease biomarkers [91]. Since multiple biomarkers play a role in the development and progression of the disease, it can be said that practical and sensitive detection methods capable of simultaneously detecting multiple biomarkers associated with a given disease (i.e., highly multiplexed detection) are urgently needed. The advantages of biomarker multiplex detection include lowering the cost of detecting a particular disease and reducing patient pain due to low sample consumption [92,93,94]. One of the main strategies that has always been considered to reduce the cost of molecular methods is the use of multiple methods or highly multiplexed analyses to simultaneously detect more than one infectious agent in a reaction [95,96,97]. In highly multiplexed analytical methods, the components and materials required for a molecular reaction are used, and only by adding additional oligonucleotides can more than one infectious agent be detected in the sample. In addition to saving financial resources, this advantage also leads to time savings [98]. More detailed information can be obtained by examining the DNA of the disease agent, because DNA contains all the genetic information of microorganisms and any other living thing. In the highly multiplexed method, the main nature of the disease is determined by examining the DNA. Therefore, to diagnose infectious diseases, the genome, which is made up of only one single-stranded RNA, is first transformed into DNA. It does this with the help of a useful enzyme called “reverse transcriptase” or “transcript”. By combining these two techniques, that is, by converting RNA to DNA and then amplifying it, the highly multiplexed method is used. Due to the existence of various information on the DNA of pathogens, the speed of treatment is increased by using highly multiplexed methods. In addition, this method can be used to obtain more accurate information about the infection. Moreover, different antibodies produce specific staining patterns on cells, depending on the cellular location and the antigenic properties of their target [56,99,100,101]. Therefore, antibodies (IgG, IgM, and IgA) against protein antigens and their components (RBD, S1, S2, S), membrane (M), and nucleoprotein (N) can be measured by high multiplexed methods. The use of highly multiplexed methods with different combinations of antibody and antigen isotopes increases the specificity and sensitivity [102,103,104,105]. Highly multiplexed assays were tested by Munro and colleagues for diagnosis on MAGPIX or Luminex instruments. The results showed that this assay not only has the same function as real-time PCR, but is also capable of detecting multiple influenza viruses [106]. In recent years, highly multiplexed biomarker detection has attracted much attention for the reasons mentioned above. Low detection limits, the ability to minimize infections, and high accuracy are some of the main advantages of plasma-engineered polymers for biomarker-based virus detection in highly multiplexed detection among diagnostic techniques [107]. Plasma-based polymers for biomarker-based virus detection are also used for multiple and selective detection of viruses, although many new methods have been optimized in assay conditions [108]. By adjusting the chemical composition and size of plasma-based polymers, plasma-based polymers for biomarker-based virus detection can be easily distinguished and identified with different emission wavelengths and excited by a single light source, indicating the many advantages of plasma-based polymers for biomarker-based virus detection in highly multiplexed detection (Figure 5) [109].

## 5. Latest Applications of Plasma-Engineered Polymer-Based Biomarker for Viral Detection

### 5.1. HIV & HCV

**Figure 6:**
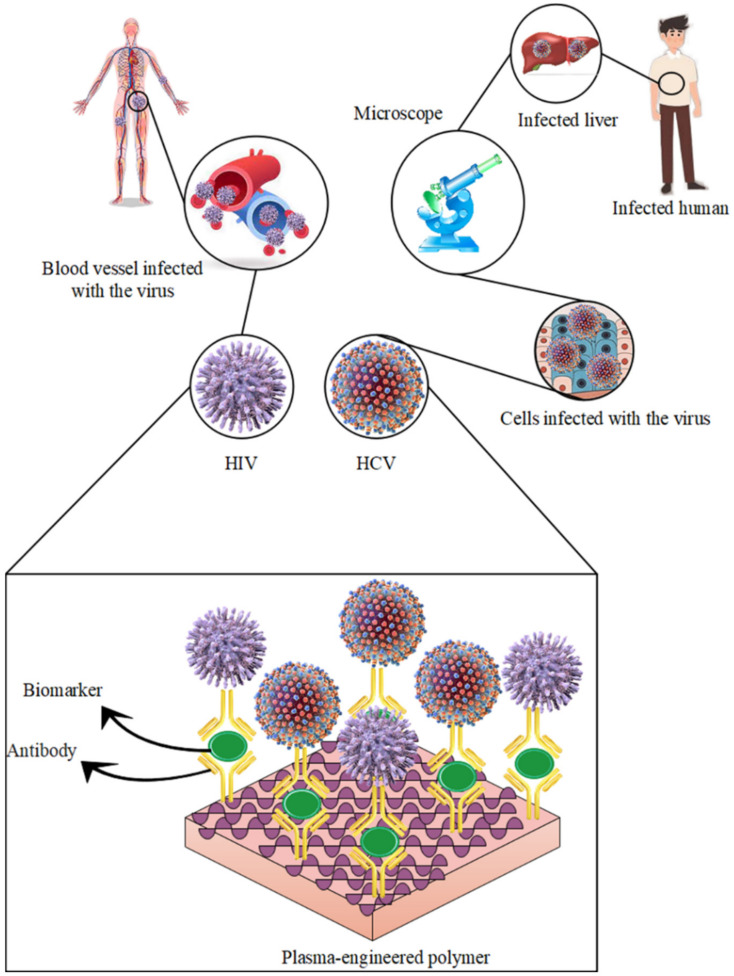
Detection of HIV & HCV by plasma-engineered polymer-based biomarker.

Human immunodeficiency virus type-1 (HIV-1), as the etiological agent of AIDS, and hepatitis C virus (HCV), as one of the most important viruses causing hepatitis, are blood-borne infectious agents. Therefore, their rapid and appropriate diagnosis in infected people is very important. In addition, ensuring the health of blood and blood products in order to prevent transmission of these viruses is a top priority of every blood-transfusion organization in the world [110,111]. As there are common routes of transmission, infection is also concurrent with HIV-1 and HCV viruses, and their prevalence is high in some community groups such as injecting drug users, hemodialysis patients, hemophiliacs and thalassemia patients. It is estimated that about ten million people worldwide are simultaneously infected with HIV-1 and HCV [112]. In a study of 499 injecting drug users in Tehran, the prevalence of co-infection with HIV-1 and HCV was estimated at 24% [113]. In another study in Lorestan province, co-infection with HIV-1 and HCV was estimated at 14.5% [114]. Concurrent HCV infection in HIV-1-positive individuals increases the risk of hepatotoxicity due to highly effective antiviral therapy and is a leading cause of death in these patients. Conversely, concurrent HIV-1 infection reduces the risk of spontaneous clearance of HCV and increases the risk of liver cirrhosis and hepatocellular carcinoma [112,115,116]. In addition to co-infection, sole infection with any of the above infectious agents is also a severe health problem due to inadequate treatment methods and the lack of effective vaccination. Therefore, the timely, accurate, and correct diagnosis of patients infected with HIV-1 and HCV viruses is very important [117]. Among the various detection methods used to identify viral infectious agents, the plasma-engineered polymer-based biomarker method (Figure 6) [118,119,120,121,122] is one diagnostic method [106,123] that is gaining more acceptance due to its sensitivity and its very practical nature, simplicity, automatability and ability to test samples in large quantities [124,125,126,127]. One of the main strategies that has always been considered to reduce the cost of detection methods is the use of plasma-based polymers for the detection of viruses based on biomarkers to simultaneously detect more than one infectious agent in one reaction [10,128,129,130].

### 5.2. COVID-19

**Figure 7:**
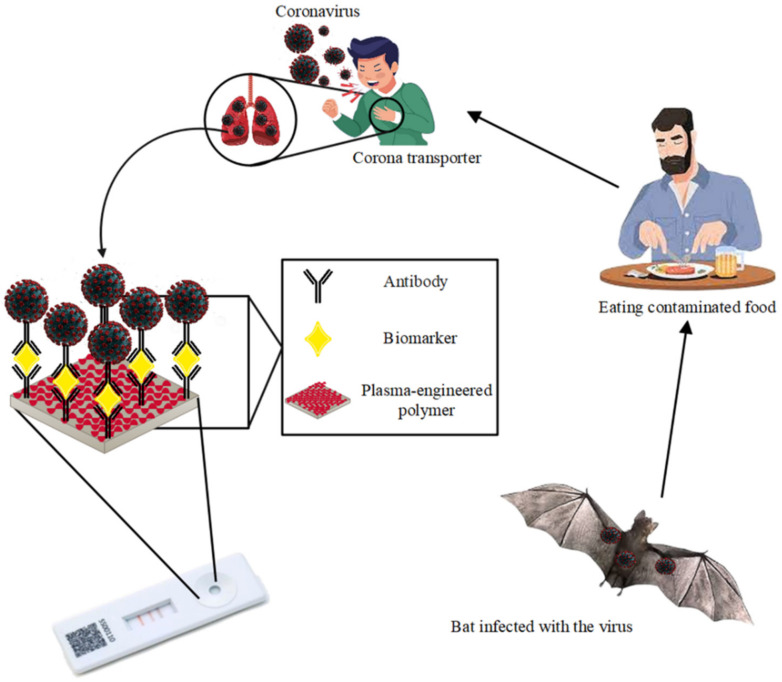
Detection of coronavirus by plasma-engineered polymer-based biomarker.

Several viruses from the coronavirus family are responsible for infectious diseases in humans and animals [131]. Coronaviruses, medium-sized, enveloped viruses and RNA have a single strand (positive strand) that spreads to humans and usually causes respiratory diseases [132]. Currently, many human coronaviruses (HCoV) such as HCoV-299E, HCoV-NL63, HCoV-HKU1 and HCoV-OC43 are the cause of mild respiratory diseases [133]. Other members of the coronavirus family, such as beta coronavirus, are transmitted from animals to humans and pose a threat to human health with outbreaks of severe respiratory diseases such as Middle East Respiratory Syndrome (MERS) and Severe Acute Respiratory Syndrome (SARS) [134]. In December 2017, an outbreak of a new coronavirus called SARS-CoV-2 was first reported in Wuhan, China, and the resulting respiratory infection was named COVID-19. COVID-19 is a respiratory illness with flu-like symptoms that manifest as a dry cough, fever, severe headache, and fatigue. People who contract COVID-19 show a wide range of symptoms from mild to severe respiratory diseases, and cases of organ dysfunction may occur, including heart damage, acute kidney damage, liver dysfunction and acute respiratory distress syndrome, which can lead to decreased lung function and cardiac arrhythmias in the long term. COVID-19 has spread rapidly worldwide, and the number of infected persons and the mortality rate has risen steadily since the outbreak [135]. Therefore, the detection of asymptomatic carriers and ill persons is important for strategies to control the epidemic. Currently, several detection methods are approved by research institutes around the world. Therefore, it can be difficult to select the optimal diagnostic method [136]. The development of new diagnostic assays that allow rapid and reliable detection of COVID-19 is critical to addressing the COVID-19 epidemic. To monitor the presence of SARS-CoV-2, it is possible to use nanotechnology-based assays as emerging innovative approaches [137,138,139]. Plasma-engineered polymer-based biomarkers may offer potential alternative approaches with low-cost, rapid, sensitive, and accurate diagnosis of the disease Figure 7 [140]. A plasma-engineered polymer-based biomarker is a way to detect a biological analyte such as a microorganism or biomolecule. Plasma-engineered polymer-based biomarkers consist of two parts: 1. target, which can be an RNA, antigen or antibody; 2. receptor-detection method, nucleic-acid probe, aptamer, and antibody [141]. In recent decades, innovation in plasma-engineered polymer-based biomarker research has seen an extraordinary and exponential increase in development and performance. Due to advances in delivery systems, nanotechnology, and genetic engineering, various strategies have been proposed to improve the detection performance of plasma-engineered polymer-based biomarkers [142].

### 5.3. Zika

**Figure 8:**
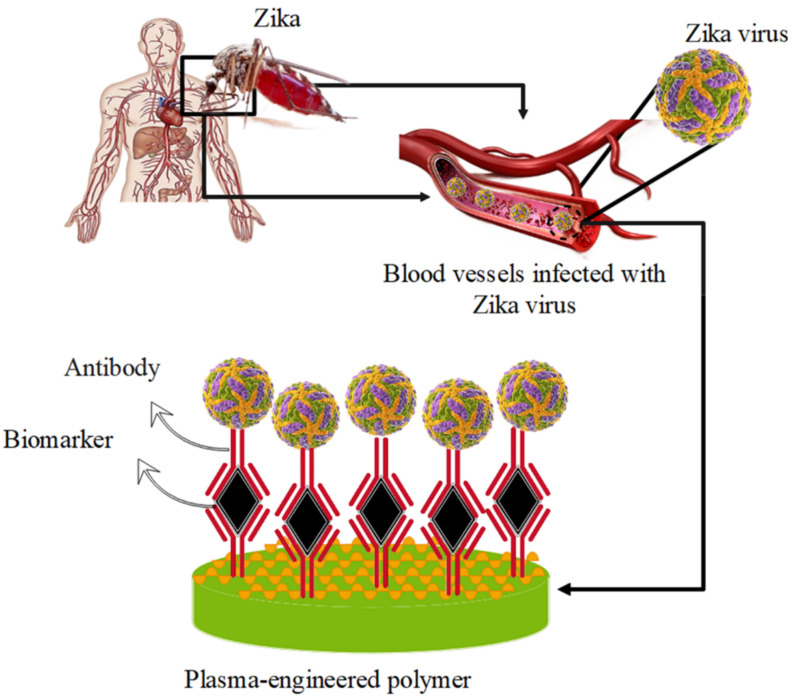
Detection of Zika virus by plasma-engineered polymer-based biomarker.

Among the emerging diseases of the 21st century, such as coronavirus and Middle East Respiratory Syndrome (MERS), Zika-virus infection has caused many public health problems and concerns worldwide. The Zika virus is a flavivirus transmitted by mosquitoes and therefore belongs to the group of arboviruses. The term arbovirus refers to viruses transmitted by arthropods and is actually the definition of viruses that are maintained in nature through biological transmission between a sensitive vertebrate host and an arthropod such as a mosquito. In previous years, before the recent Zika-virus outbreak [143,144,145], there was little concern about this viral infection, since the virus was associated with mild fever, rash and joint pain in only 20% of cases, and it did not show any recognizable clinical symptoms in 80% of cases [146]. However, recent widespread outbreaks of the virus have occurred in the Pacific region, notably on Yap Island in 2007 and in French Polynesia in 2013. In 2014, cases of Zika-virus infection were reported on Easter Island in the Pacific, which was part of the country of Chile. Subsequently, from 2015 to 2016, a greater spread of this viral infection was observed in South and Central America, particularly in Brazil, prompting the World Health Organization to declare a state of emergency for this infection [147,148]. Plasma-engineered polymer-based biomarkers can be a valuable alternative for detection. Polymer-based biomarkers are usually developed using a biological component with an engineered plasma [149]. A major goal of the plasma-engineered polymer-based biomarker method is to (a) retain the best features of traditional detection methods, which are highly sensitive and specific, and (b) enable cost-effective methods that can be used more accurately and non-invasively with real-time detection [64,150,151]. However, few plasma-engineered polymer-based biomarkers for Zika-virus detection are characterized by these features, as most detect different Zika-virus antigens with conventional monoclonal antibodies cast on a specific platform (Figure 8). This leads to methods with limited selectivity in terms of the specificity of the antibodies used. In this context, the development of a polymer-based biomarker for the early detection of the Zika virus has been considered based on the high sensitivity provided by plasma-engineered polymers [152].

### 5.4. Influenza

**Figure 9:**
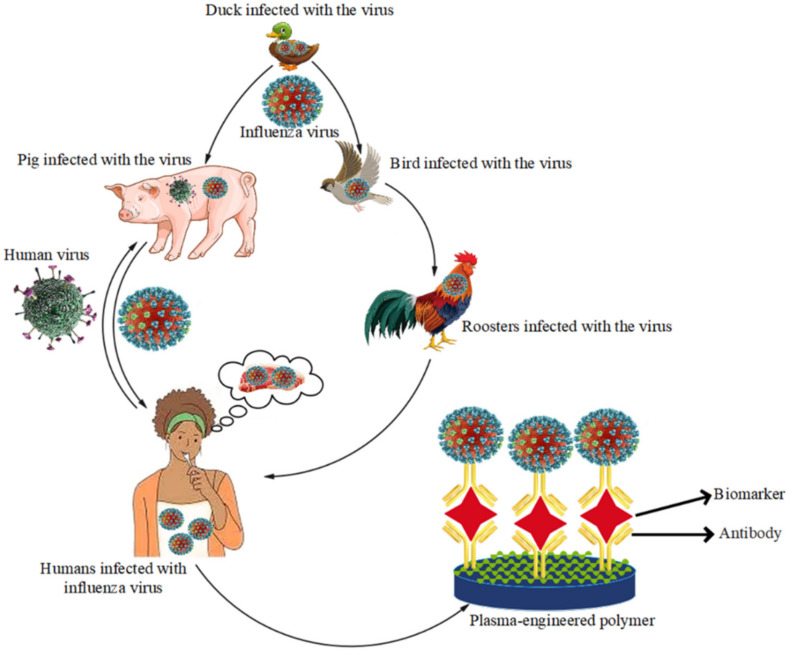
Detection of influenza virus by plasma-engineered polymer-based biomarker.

The polymorphic, spherical virions of the family Orthomyxoviridae have a diameter of 80–120 nm and possess a single-stranded genome of negatively polar, fragmented RNA and a lipid envelope. Thus, the influenza virus is an RNA virus of the Orthomyxoviridae family that causes acute respiratory infections and influenza or Grippe contagious disease [153]. Influenza viruses A, B, and C are classified into three types based on the antigenic differences of the matrix proteins (M) and the nucleoprotein (NP). To date, the type A virus has been divided into 17 different hemagglutinin subtypes of hemagglutinin (H1 to H17) and 10 different neuraminidase subtypes (N1 to N10) based on the surface antigens of hemagglutinin (HA) and neuraminidase (NA) [154,155]. Influenza A viruses (IAVs) are genetically diverse pathogens whose genome changes, and due to changes in the structure of the viral surface proteins, new types with different characteristics from the previous virus emerge every few years [156]. In humans, only influenza A and B viruses are epidemiologically considered [156,157]. The emergence of subtypes H5N1, H7N9, and subtype of influenza A virus H9N2, as well as the increase in genetic exchange between influenza viruses in birds and poultry (H5N1-/A, Avian) [158,159], wild waterfowl, domestic birds, pigs [160], and humans, have been introduced as a constant threat to humans [161]. There was another pandemic similar to this one in 1918, which claimed 50 million lives worldwide. In the twentieth century, there were three influenza pandemics, each of which created a human viral strain that killed tens of millions of people [162,163,164]. All subtypes of influenza A have previously been isolated from birds, but subtypes H2N2, H1N1, and H3N2 have only been isolated from human communities in 1957, 1918, and 1968, respectively [165,166,167]. The emergence of new strains through mutation and reassortment of gene fragments between different human and animal strains allows for different subtypes of the influenza A virus [168,169]. The first report of the influenza A virus outbreak in Iran was published in 2010. This identified the rearrangement of genome 3 of the human, porcine and avian influenza virus, now known as influenza A/H1N1 virus. Plasma-engineered polymer-based biomarkers offer the advantages of ease of use, rapid response, low cost, portability, automation, and ease of integration with nanomaterials, polymers/nanostructures, and universal biotechnology. One of the most important requirements in developing a plasma-engineered polymer-based biomarker for influenza viruses is the need for a sensitive analytical method that can easily reduce very low limits of detection without significant changes in selectivity. Influenza viruses spread rapidly in a community before symptoms appear for identification. A plasma-engineered polymer-based biomarker that can detect the target virus rapidly, sensitively, and selectively will be invaluable. In addition, a simple, strong, rapid, and cost-effective plasma-engineered polymer-based biomarker is urgently needed for this field. While polymer-based biomarkers have been developed for a variety of applications, plasma-based polymers have been used for biomarker-based virus detection (Figure 9) [170,171,172,173,174].

## 6. Future and Perspectives

Plasma-engineered polymer-based biomarkers must be robust and reproducible in order to become the primary method for detecting pandemic viruses. Thus, plasma-engineered polymer-based biomarkers can significantly help prevent future epidemics by providing faster and more accurate detection of pandemic viruses. For this group of biomarkers, the mechanism of the studied plasma-engineered polymer-based biomarkers is still valid. As their diagnosis becomes more frequent and significant worldwide, this is particularly important for non-communicable diseases. Most importantly, highly multiplexed analysis is possible as a new type of simple and flexible approach for new detection technologies and intracellular probes. Thus, this review study opens a new avenue for recent advances in plasma-engineered polymers for biomarker-based virus detection and highly multiplexed analysis.

## 7. Conclusions

With the increasing number of patients with infectious diseases and the resulting mortality, treatment is becoming a very important issue for prevention, which also depends on early and accurate diagnosis. Therefore, much research has been performed in the field of detection and prediction of difficult diseases by biomarkers because they are credible and provide valuable help, despite the many problems in the field of biomarkers. The availability of accurate and effective polymer-based biomarkers enables the diagnosis and classification of diseases. The development of new plasma-engineered polymer-based biomarkers has also attracted much attention. Therefore, studies have been conducted on the efficacy of polymer-based biomarkers for the diagnosis and prediction of the use of polymer-based biomarkers in the development of effective detection against pandemic viruses. Recently, plasma-engineered polymers, the processing of polymer surface morphology in plasma, and the processing of polymers in plasma surface chemistry have been particularly useful for this purpose.

## Figures and Tables

**Table 1:** Plasma-engineered polymer-based biomarker developed to detect biomarkers of viral diseases.

Infectious Disease	Infectious Biomarker	Detection Techniques	Ref.
Hepatitis B	AFP	ELISA	[25]
Hepatitis B	DCP	Electrochemiluminescence immunoassay	[26]
Hepatitis B	miRNA-21	qRT-PCR	[27]
HIV	microRNA	RT-qPCR	[28]
HIV	HIV RNA	Cerebrospinal Fluid (CSF)	[29]
HIV	HIV antibodies	Plasma	[30]
COVID-19	Viral RNA Genome	PCR Point-of-care detection	[31]
COVID-19	Spike Protein	ELISA Laboratory testing	[32]
ZIKV	IgM antibodies	RT-PCR	[33]
Influenza	miRNAs	RT-*q*PCR	[34]
Influenza	719 DEGs	Weighted gene co-expression network analysis (WGCNA)	[35]

## Data Availability

All data generated or analyzed during this study are included in this published article.
